# Persistently ambiguous: a taxometric investigation on two groups of suicidal ideation indicators

**DOI:** 10.3389/fpsyg.2026.1729597

**Published:** 2026-02-25

**Authors:** Lucas de Francisco Carvalho, Gisele Magarotto Machado, Giselle Pianowski, Nelson Hauck-Filho, Makilim Nunes Baptista, Cato Grønnerød

**Affiliations:** 1 Department of Psychology, University of São Francisco, Campinas, Brazil; 2 Division of Mental Health Services, R&D Department, Akershus University Hospital, Oslo, Norway; 3 Department of Psychology, PROMENTA Research Center, University of Oslo, Oslo, Norway; 4 Pontifical Catholic University of Campinas (PUC-Campinas), School of Life Sciences, Postgraduate Program in Psychology, Campinas, Brazil

**Keywords:** mental disorders, psychopathology, public health, risk behavior, self-harm

## Abstract

**Introduction:**

We performed a taxometric investigation into the underlying structure of suicidal ideation in two diverse samples of nonclinical adult populations.

**Methods:**

We relied upon one sample of 547 individuals, aged 18–78, who responded to the Beck Scale for Suicide Ideation (BSS), and one sample of 989 participants aged 18–64 who responded to the Suicidal Ideation Attributes Scale (SIDAS). We analyzed the data using different taxometric techniques: MAMBAC, MAXEIG, and L-Mode.

**Results:**

Our findings suggest ambiguity in the suicidal ideation latent structure (Mean CCFI BSS dataset = 0.46; Mean CCFI SIDAS dataset = 0.45) the results neither indicate any clear tendency toward taxonicity nor toward dimensionality.

**Conclusion:**

We discuss them based on the possibility that suicidal ideation may represent a complex construct encompassing multiple components.

## Introduction

Suicidal behavior is one of the most critical and complex concepts associated with mental disorders. Approximately 800,000 deaths by suicide occur worldwide each year (World Health Organization, 2021). A substantially larger number of individuals experience suicide attempts, and an even greater proportion report suicidal ideation, a common yet difficult-to-predict phenomenon. Suicidal behavior encompasses a range of experiences, including passive and active ideation, attempts, and potential fatality as outcomes (Gvion and Apter, 2012). This complex phenomenon is prevalent across different demographic groups and is characterized by a multitude of risk and protective factors (Beghi et al., 2021; Evans et al., 2017; Nock et al., 2008). Despite ongoing efforts to understand the profiles of those at risk for suicide, accurately predicting suicide remains challenging (Belsher et al., 2019), due to the multitude of factors that influence risk. Previous suicide attempts and suicidal ideation and are key risk factors for suicide behavior and potential fatality, making them central targets in psychology research (Baptista et al., 2022; Harmer et al., 2022; May and Victor, 2018; Turecki et al., 2019).

There is still an ongoing debate concerning the latent structure of suicidal ideation, as evidence from prior research remains inconsistent regarding whether this phenomenon is best conceptualized as categorical or dimensional. Some studies have reported findings suggestive of a categorical latent structure (e.g., Orlando et al., 2015), whereas others have provided evidence in favor of dimensional models (e.g., Liu et al., 2015). Despite numerous investigations, this question remains unresolved. The present study aims to conduct a taxometric investigation of the latent structure of suicidal ideation in two non-clinical adult samples to contribute to this debate.

Classification systems are essential tools that help health professionals interpret the existence of symptoms and signs of various mental disorders and communicate in a universal technical language (Kendler, 2009). Since the first version of the Diagnostic and Statistical Manual of Mental Disorders (DSM; American Psychiatric Association, 1952), there has been a concerted effort to categorize mental health conditions. This initiative aims to facilitate the collection of reliable information about individuals in psychological assessment contexts and facilitate global exchange by establishing organized criteria for diagnoses, which were – and still are – primarily categorical, focusing on the presence or absence of a disorder.

However, more recent approaches have emphasized the need to rethink the comprehension of mental disorders based on dimensional models (Conway et al., 2019; Hopwood et al., 2018). These models guide the understanding of disorders through the quantitative gradation of symptoms, which can vary in intensity on a continuum that ranges from healthy to pathological (American Psychiatric Association, 2022; Kotov et al., 2017). Several researchers favor the dimensional perspective on the taxonomy of mental disorders (Brown and Barlow, 2005; Krueger and Bezdjian, 2009; Widiger and Samuel, 2005). Achenbach and Edelbrock (1983) were precursors of dimensional approaches, indicating that assessment scales consistent with dimensional proposals could favor a more complete and qualitative view of human phenomena. That is, they could provide enhanced clinical information (Achenbach et al., 2016). More recently, dimensional proposals gained prominence with recent representatives as the National Institute of Mental Health’s Research Domain Criteria (RDoC; Insel et al., 2010), the Hierarchical Taxonomy of Psychopathology (HiTOP; Kotov et al., 2017), the International Statistical Classification of Diseases and Related Health Problems (ICD-11; World Health Organization, 2018), and the DSM-5 alternative model for personality disorders (American Psychiatric Association, 2013).

Researchers have used taxometric procedures to investigate the latent structure of several psychopathologies. Understanding whether the latent structure of psychopathology is more suitable for categorical or dimensional models, can guide research and favor clinicians in their evaluations and interventions. Meta-analytical studies demonstrate the latent structures of most mental disorders are better explained by dimensional models (Haslam et al., 2012). In a more recent meta-analysis, Haslam et al. (2020) similarly found that the majority of the investigated constructs and psychopathologies show evidence of dimensionality. However, in this meta-analysis, suicide risk was among the few psychopathologies in which results suggested plausible taxonicity (i.e., categorical latent structure). Despite this, the authors noted that the plausibility level was not very high, as the taxonic results were replicated only a single time. Moreover, in that review, suicide risk was operationalized broadly, encompassing heterogeneous indicators, including suicidal ideation, suicidal self-injury, and suicide risk under the same umbrella. A more specific meta-analysis focused exclusively on suicidal ideation reported a mean CCFI value of 0.48, indicating an ambiguous latent structure (Siddaway et al., 2021). This meta-analysis revealed mixed findings across studies, with some suggesting dimensionality and others taxonicity. The authors further emphasized the limited number of available studies and the inconsistency of the results, concluding that it is premature to draw definitive conclusions regarding the latent structure of suicidal thoughts and that additional research is needed to clarify this issue.

Determining the latent structure of suicidal ideation is critical for understanding whether individuals experiencing suicidal thoughts should be conceptualized as belonging to a distinct subgroup (i.e., a taxon) or whether suicidal ideation is better understood as varying continuously in severity across individuals. These alternative conceptualizations have important implications for clinical practice, particularly with respect to the use of diagnostic thresholds, risk stratification, intervention, and prevention decisions. A categorical model emphasizes the identification of cutoffs to distinguish individuals who “have” versus “do not have” suicidal ideation, whereas a dimensional model suggests that suicidal thoughts may be present to varying degrees and warrant clinical attention across the full continuum (Haslam et al., 2020). Moreover, latent structure has implications beyond cross-sectional classification. A dimensional structure would suggest that suicidal ideation reflects a gradual accumulation of risk, supporting models that emphasize early identification and intervention even at lower levels of ideation severity. In contrast, a categorical structure would imply a qualitative shift in psychological functioning, consistent with threshold-based identification of a high-risk subgroup. Clarifying this distinction may also inform efforts to differentiate individuals who experience suicidal ideation but never attempt suicide from those who progress to suicidal behavior (Gvion and Apter, 2012; Klonsky et al., 2018), thereby contributing to more precise risk stratification and targeted prevention strategies.

### The current study

We aimed to perform a taxometric investigation into the underlying structure of suicidal ideation in two diverse samples of nonclinical adult populations. We used six variables (i.e., scores) from the Beck Scale for Suicide Ideation (BSI; Beck et al., 1979) and the Suicidal Ideation Attributes Scale (SIDAS; Van Spijker et al., 2014) representing the wish for death, suicide ideation, interference in daily activities due to suicidal thoughts, preparation for suicide, active suicide desire, and proximity to a suicide attempt. Given the fragmented and conflicting evidence on taxononicity versus dimensionality, we believe there remains a need to investigate the underlying structure of suicidal ideation.

## Materials and methods

### Participants

We conducted this study with two separate data collections. The first comprised 2,014 individuals, aged 18–90 (*M* = 30.58; SD = 12.55), who responded to the Beck Scale for Suicide Ideation (BSS). The majority were women (51.6%), single (52.2%), Caucasian (51.2%), and with complete high school (36.4%). In sum, 17.23% of the respondents self-reported having attempted suicide at some point in their lives, and 42.6% reported a history of or current suicidal ideation.

The BSS includes two screening items within the first five items; respondents who score 0 on both are usually instructed to skip the remaining items and proceed directly to the final item. In the present data collection, however, participants were instructed to complete all items regardless of their responses to these screening items. Nevertheless, to address potential floor effects associated with this skip structure, we excluded participants who scored 0 on both screening items. This procedure resulted in a sample of 547 participants aged 18–78 years (*M* = 26.03, SD = 8.22). In this sample, the majority of participants were women (51.2%), single (62.3%), identified as Caucasian (47.7%), and reported having completed high school (40.9%). Within this group, 38.4% self-reported a lifetime history of suicide attempts, and 87.7% reported a history of or current suicidal ideation, reflecting the exclusion of participants without endorsed ideation.

The second sample comprised 989 participants aged 18–64 (*M* = 20.07; SD = 5.02) who responded to the Suicidal Ideation Attributes Scale (SIDAS). The second sample was primarily women (87.1%), single (91.8%), Caucasian (55.7%), and with complete high school (64.9%). A total of 49.8% self-reported attempting or thinking about attempting suicide at some point in their lives. Although detailed information regarding the suicide attempts was not available for either sample, the self-reported information suggests variability in the psychopathology levels of our samples, despite the sample being drawn from the general population.

### Measures

#### Beck Scale for Suicide Ideation (BSS)

The BSS (Beck et al., 1979, 1996) is a self-reported measure designed to assess suicidal ideation and a broad set of closely related suicidal attitudes and behaviors. The BSS comprised 21 items, of which 19 contribute to score computation. The items must be answered on a scale ranging from 0 to 2. Within the first five items, the BSS includes two screening items; respondents who score 0 on both are typically instructed to skip the remaining items and proceed directly to the final item. In the present study, participants were instructed to complete all items regardless of their responses to the screening items, deviating from the standard skip instruction. We calculated the BSS scores based on the three-factor solution reported in previous studies (Alsalman and Alansari, 2017; Steer et al., 1993), which are grouped into the following factors: Wish for Death, Preparation for Suicide, and Active Suicide Desire. The decision to rely on a three-factor solution for the BSS, rather than the traditional one-factor solution, was driven by the methodological requirements of taxometric analyses, which require at least three valid indicators. In our sample, Cronbach’s alpha for the factors varied from 0.85 to 0.91, while McDonald’s omega varied from 0.86 to 0.92.

#### Suicidal Ideation Attributes Scale (SIDAS)

The SIDAS (Van Spijker et al., 2014) is a self-report measure developed to assess five components of suicidal ideation: frequency, controllability, closeness to attempts, distress, and interference with daily activities. The scale comprises five items, each designed to capture one of the suicidal ideation components described. The items must be answered on an 11-point scale, varying in intensity from 0 to 10. Previous studies support the adequacy of SIDAS (Gauvin et al., 2021; Harris et al., 2021). The scale’s internal consistency was good (α = 0.81; ω = 0.86) in our sample.

### Procedures

This study adhered to the ethical research principles of the Declaration of Helsinki (World Medical Association, 2013), and was approved by a Brazilian research ethics committee (Universidade São Francisco – CAAE: 94975418.4.0000.5514).

Data were collected online between June and December 2021 using Google Forms for both data collections. Participants were recruited by sharing the study link via Facebook, WhatsApp, and other social media platforms. The link was disseminated both through personal networks of the authors and through paid Facebook advertisements to reach individuals outside these networks. In addition, a snowball sampling strategy was employed to increase the number of participants. The online survey followed recommended standards for conducting and reporting web-based surveys, specifically the Checklist for Reporting Results of Internet E-Surveys (CHERRIES; Eysenbach, 2004). All participants provided informed consent prior to participation. No incentives (monetary or otherwise) were offered for participation.

### Data analysis

We performed a taxometric analyses using two sets of suicidal ideation indicators. In our study, an indicator refers to a score used in the taxometric analyses. For the BSS dataset, the indicators were the scores on the BSS three factors: Wish for Death, Preparation for Suicide, and Active Suicide Desire. For the SIDAS dataset, the indicators were the scores on each of the five items that comprise the SIDAS scale. Taxometric analyses require a minimum of three valid indicators in each dataset. An indicator is considered valid when it can discriminate between the extremes of the sample with an effect size of Cohen’s *d* ≥ 1.25 (Meehl, 1992). Accordingly, we first investigated the effect sizes for each indicator of both datasets using Cohen’s *d*. All indicators in the BSS dataset presented adequate *d*-values. In the SIDAS dataset, items 1 and 2 did not reach the minimum *d*-values to be considered valid indicators; therefore, we excluded them from the subsequent analysis. Although taxometric analyses do not require normally distributed indicators, substantial deviations from normality, particularly pronounced skewness, have been shown to affect taxometric results (Ruscio et al., 2004). Therefore, we also examined descriptive statistics (mean, standard deviation, skewness, and kurtosis) for all indicators in both datasets.

After establishing the quality of the indicators, we conducted three different nonredundant taxometric methods, as recommended in previous studies (Meehl, 1992; Ruscio et al., 2010). We employed the following methods in our study: Mean Above Minus Below a Cut (MAMBAC; Meehl and Yonce, 1994), Maximum Covariance (MAXEIG; Meehl and Yonce, 1996), and Latent Mode Factor Analysis (L-Mode; Waller and Meehl, 1998). All methods were based on the data comparison procedure (Meehl, 1995), which allows the empirical curves to be compared against prototypal curves of simulated (a) dimensional and (b) taxonic data that resemble the descriptive characteristics of the researcher’s data (Ruscio et al., 2010). The simulated data keep the same characteristics as the empirical data (e.g., mean and standard deviation), differing only in terms of the latent structure (Ruscio and Ruscio, 2004).

Additionally, we inspected the Comparison Curve Fix Index (CCFI) values of all the methods. The CCFI is calculated by estimating the distance between the curves generated for the empirical and the simulated data. CCFI can vary from 0 to 1, with values closer to 0 representing evidence of dimensionality and values closer to 1 indicating taxonicity. Values between.40 and.60 are considered ambiguous, not allowing conclusions regarding the latent structure of the variable being studied (Ruscio et al., 2018). Ruscio et al. (2018) recommend using the mean CCFI value (mean of CCFI across different methods) to interpret the taxometric analysis results. Given that all taxometric methods require the specification of a taxon base rate (i.e., the proportion of individuals in the sample assumed to belong to a taxon, which was specified as 25% in our study), and that this base rate is an assumption that may be unrealistic, particularly when the underlying structure is dimensional and no true base rate exists, we additionally generated CCFI Profiles for both datasets. CCFI Profiles were obtained by applying the taxometric methods MAMBAC, MAXEIG, and L-Mode across a range of assumed taxon base rates, from 0.025 to 0.975 in increments of 0.025. For each base rate and method, a CCFI value was computed, along with aggregate CCFI values for each method and an overall mean CCFI across methods (Ruscio et al., 2018). We performed all the analyses on R software using the RTaxometrics package (Ruscio and Wang, 2021).

Because analytic decisions regarding indicator selection and participant exclusion may influence the results, we conducted sensitivity analyses for both datasets. For the BSS dataset, analyses were repeated without excluding participants who scored 0 on both screening items, as well as with a base-10 logarithmic transformation applied to the data, given that the raw BSS indicators showed substantial deviations from normality. For the SIDAS dataset, analyses were repeated without excluding Items 1 and 2. Additional details and results from the sensitivity analyses are reported in the Supplementary materials.

## Results

Before proceeding with the taxometric analyses, we assessed the validity and adequacy of the indicators. Table 1 presents the validity (Cohens *d*) and descriptive statistics (mean, standard deviation, skewness, and kurtosus) of the indicators in both the BSS and the SIDAS datasets. All indicators of both datasets obtained adequate skewness and kurtosis estimates. Cohen’s *d*-values were acceptable (≥1.25) for all indicators in the BSS dataset. In contrast, two indicators in the SIDAS dataset (items 1 and 2) fell below the required threshold and were therefore excluded from subsequent analyses. Consequently, taxometric analyses for the SIDAS dataset were conducted using items 3 through 5.

**TABLE 1:** Descriptive and quality of the indicators.

BSS dataset (*N* = 547)				
	*M* (SD)	Skewness	Kurtosis	*d*
Wish for death	7.12 (3.13)	0.22	−0.20	2.07
Preparation for suicide	2.68 (2.67)	1.05	0.66	2.36
Active suicide desire	2.00 (1.79)	1.17	0.85	2.57
**SIDAS dataset (*N* = 989)**				
	*M* (SD)	Skewness	Kurtosis	*d*
Frequency of thoughts about suicide (SIDAS 1)	4.91 (3.68)	0.42	−1.33	0.31
Control over suicidal thoughts (SIDAS 2)	5.53 (3.76)	−0.17	−1.51	−0.01
Closeness to attempt (SIDAS 3)	2.15 (3.16)	1.29	0.23	2.75
Distress (SIDAS 4)	4.01 (4.07)	0.42	−1.52	2.25
Interference with daily activities (SIDAS 5)	3.19 (3.77)	0.77	−1.02	3.67

BSS, Beck Scale for Suicide Ideation; SIDAS, Suicidal Ideation Attributes Scale; M, mean; SD, standard deviation; *d* = Cohen’s *d* effect size.

**FIGURE 1:**
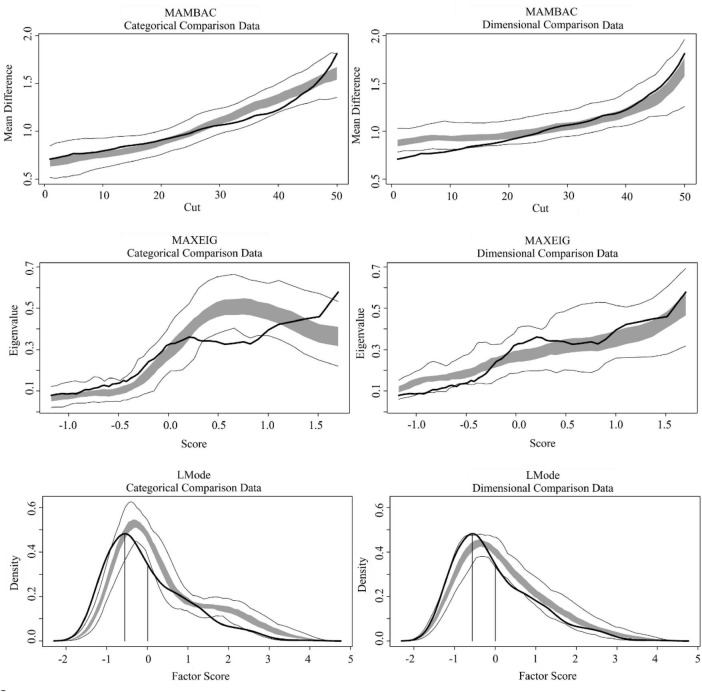
Taxometric results BSS dataset. BSS, Beck Scale for Suicide Ideation. The solid black line represents values computed from the observed data. The dark gray band represents values obtained from simulated taxonic (left panel) and dimensional (right panel) comparison data. Light gray lines represent variability across the simulated datasets. Base-rate estimates derived from each method were as follows: MAMBAC = 0.31, MAXEIG = 0.15, L-Mode = 0.62, and mean = 0.36. As expected under ambiguous solutions, estimates the derived base-rates estimates varied substantially across methods.

**FIGURE 2:**
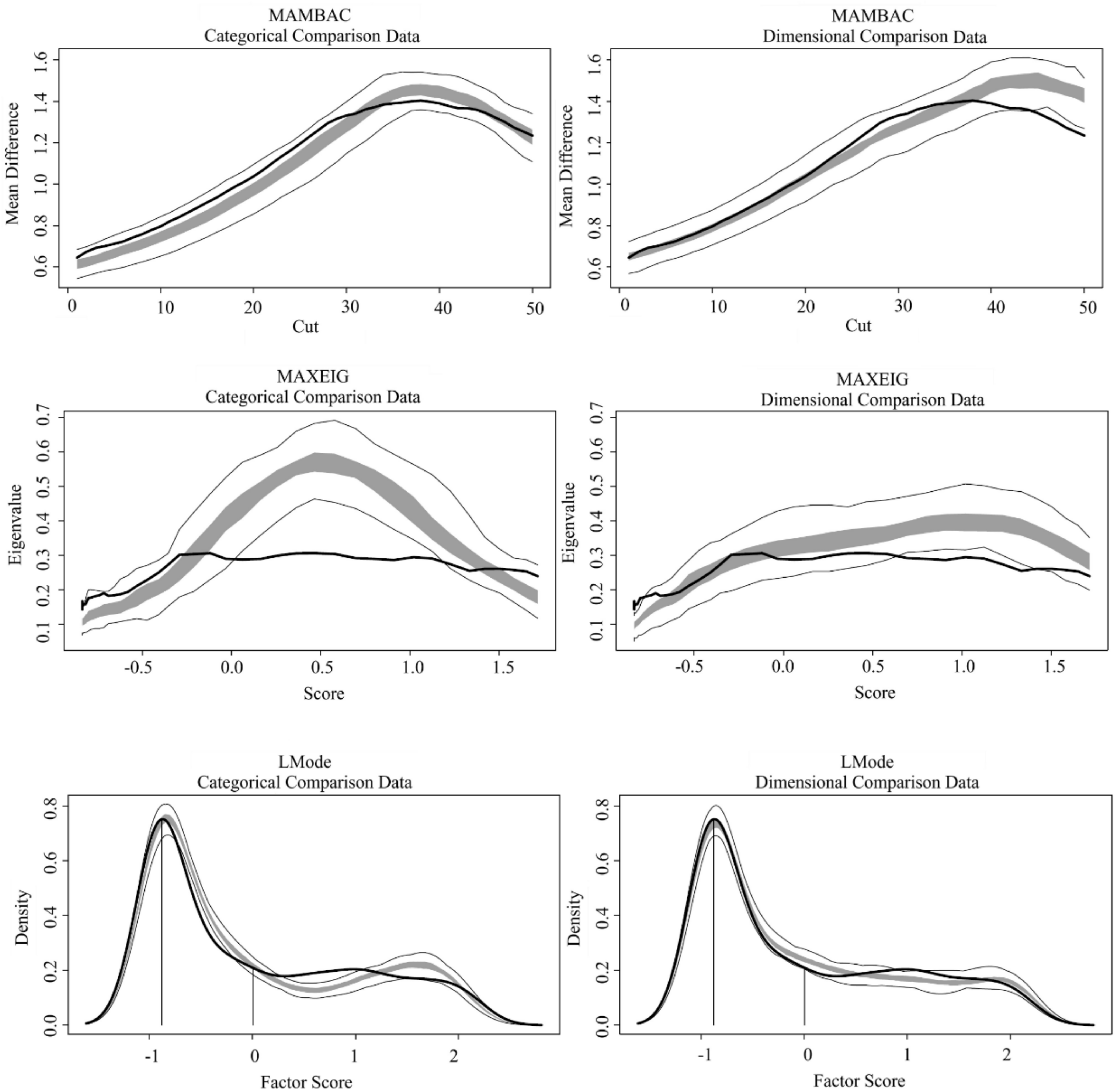
Taxometric results SIDAS dataset. SIDAS, Suicidal Ideation Attributes Scale. The solid black line represents values computed from the observed data. The dark gray band represents values obtained from simulated taxonic (left panel) and dimensional (right panel) comparison data. Light gray lines represent variability across the simulated datasets. Base-rate estimates derived from each method were as follows: MAMBAC = 0.34, MAXEIG = 0.21, L-Mode = 0.72, and mean = 0.42. As expected under ambiguous solutions, estimates the derived base-rates estimates varied substantially across methods.

After attesting the adequacy and validity of the indicators, we proceed with the taxometric analyses. Figures 1, 2 depict the taxometric analyses results for the BSS and SIDAS datasets, respectively. Table 2 presents the CCFI values for the three taxometric methods used in this study (MAMBAC, MAXEIG, and L-Mode), as well as the mean CCFI across methods.

**TABLE 2:** Results of taxometric analysis.

Dataset	CCFI by method			
	MAMBAC	MAXEIG	L-Mode	Mean CCFI
BSS dataset	0.58	0.36	0.44	0.46
SIDAS dataset	0.59	0.36	0.39	0.45

BSS, Beck Scale for Suicide Ideation; SIDAS, Suicidal Ideation Attributes Scale; CCFI, Comparison Curve Fix Index; MAMBAC, Mean Above Minus Below a Cut; MAXEIG, Maximum Covariance; L-Mode, Latent Mode Factor Analysis.

For all taxometric methods, the gray lines in Figures 1, 2 represent the shape of the simulated taxonic and dimensional data, whereas the solid black line represents the empirical data. Visual inspection of the BSS results suggests that, in the MAMBAC method, the empirical curve more closely resembles the simulated taxonic curve than the simulated dimensional curve. In contrast, for MAXEIG and L-Mode, the empirical curves appear more similar to the simulated dimensional curves. A comparable pattern was observed for the SIDAS dataset: visual inspection suggests greater similarity to the simulated taxonic curve in MAMBAC, but closer resemblance to the simulated dimensional curves in MAXEIG and L-Mode. However, visual inspection is inherently limited by its subjectivity and the absence of objective thresholds for determining the degree of similarity between empirical and simulated curves. Therefore, we relied primarily on the CCFI values to interpret the results. As shown in Table 2, the CCFI values were generally consistent with the visual impressions, with MAMBAC suggesting a structure closer to dimensionality and MAXEIG and L-Mode suggesting a structure closer to taxonicity across both datasets. Nevertheless, most CCFI values (except for MAXEIG) fell within the ambiguous range (0.40–0.60), impeding definitive conclusions regarding the latent structure. Importantly, the mean CCFI values also fell within this ambiguous interval, with values of 0.46 for the BSS dataset and 0.45 for the SIDAS dataset, further supporting an interpretation of structural ambiguity.

**FIGURE 3:**
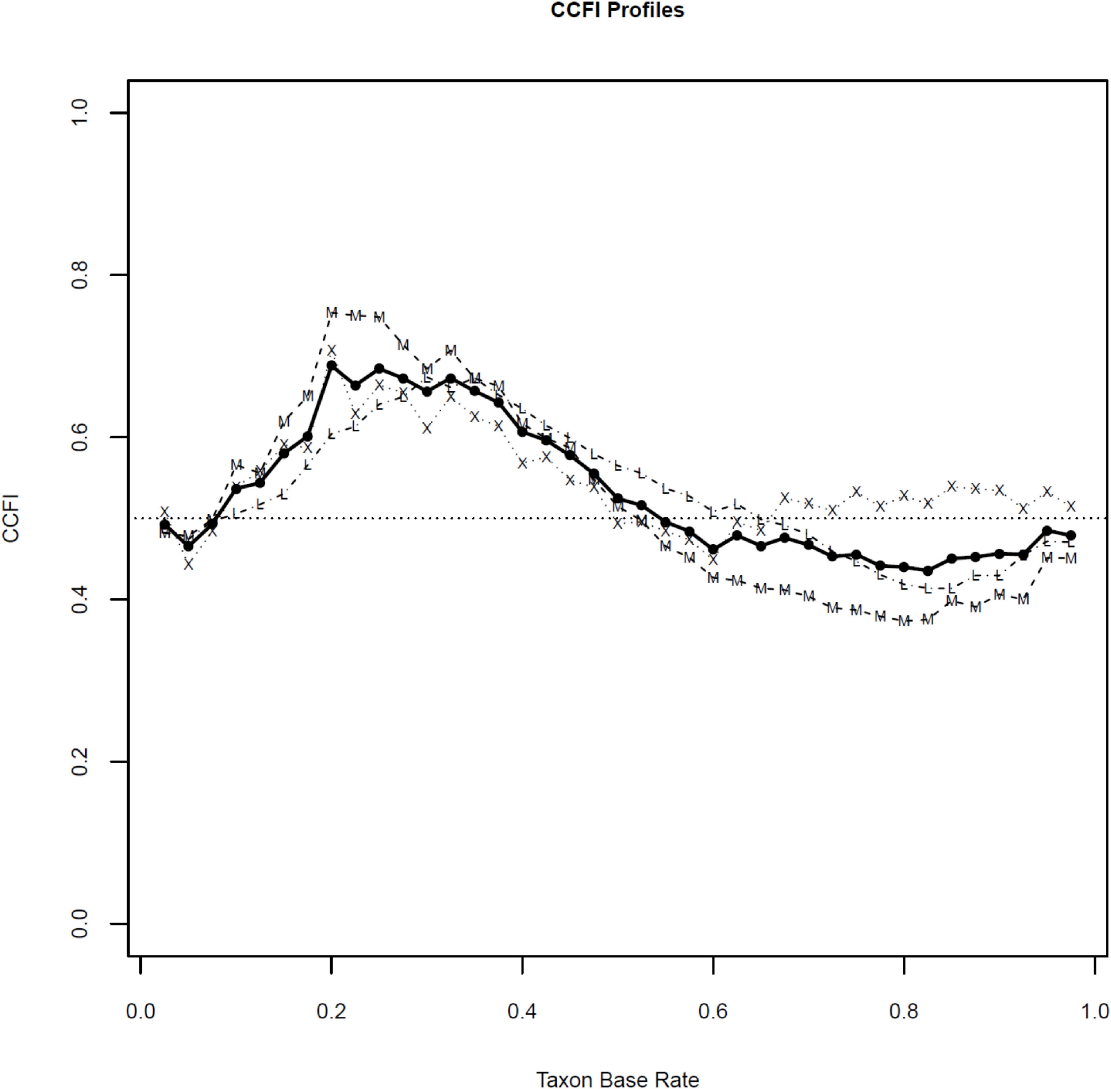
CCFI profiles BSS dataset. BSS, Beck Scale for Suicide Ideation; CCFI, Comparison Curve Fix Index. CCFI profiles were generated across assumed taxon base rates ranging from 0.025 to 0.975 in increments of 0.025. Dotted lines with “M” represent CCFI values derived from MAMBAC, dotted lines with “X” represent CCFI values derived from MAXEIG, and dotted lines with “L” represent CCFI values derived from L-Mode. The solid black line represents the aggregated CCFI value across methods.

**FIGURE 4:**
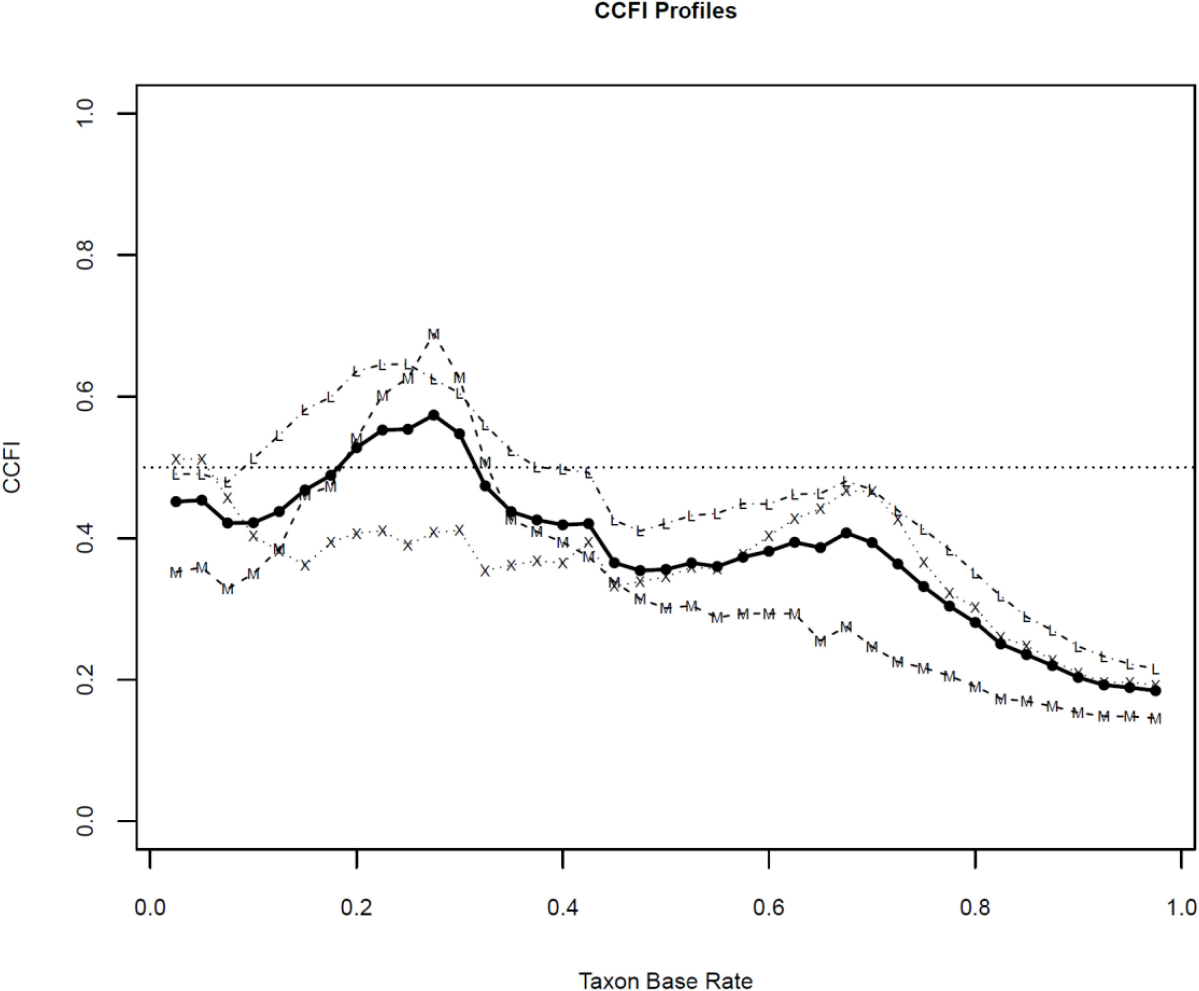
CCFI profiles SIDAS dataset. SIDAS, Suicidal Ideation Attributes Scale; CCFI, Comparison Curve Fix Index. CCFI profiles were generated across assumed taxon base rates ranging from 0.025 to 0.975 in increments of 0.025. Dotted lines with “M” represent CCFI values derived from MAMBAC, dotted lines with “X” represent CCFI values derived from MAXEIG, and dotted lines with “L” represent CCFI values derived from L-Mode. The solid black line represents the aggregated CCFI value across methods.

To further examine the latent structure of the data, we generated CCFI profiles, which display CCFI values derived from MAMBAC, MAXEIG, and L-Mode across a range of putative taxon base rates. Figures 3, 4 present the CCFI profiles for the BSS and SIDAS datasets, respectively.

In both cases, visual inspection of the profiles indicates that CCFI values across most putative base rates predominantly fall within the ambiguous range for both datasets. More importantly, the aggregate CCFI values also remained within this ambiguous interval. For the BSS dataset, the mean aggregate CCFI profile was 0.57, with method-specific values of 0.58 for MAMBAC, 0.57 for MAXEIG, and 0.57 for L-Mode. For the SIDAS dataset, the mean aggregate CCFI profile was 0.43, with corresponding values of 0.40 for MAMBAC, 0.40 for MAXEIG, and 0.51 for L-Mode.

## Discussion

We aimed to investigate the nature of the latent structure of suicidal ideation. To contribute with empirical evidence to the ongoing debate about this topic, we leveraged data from two psychological instruments assessing suicidal ideation and two distinct nonclinical adult samples to taxometrically examine the latent structure of suicidal ideation. However, the taxometric ambiguity that sustains discussions about suicidal ideation also permeated our results, which were inconclusive. For both the BSS and SIDAS datasets, results consistently fell within the ambiguous interval. This pattern also persisted when testing the robustness of the findings through sensitivity analyses involving alternative methodological choices. The uncertain taxonicity or dimensionality of suicidal ideation poses two venues of discussion, one about the suicidal ideation itself, and the other about the applicability of the taxometric analysis. We prompt researchers to exercise caution in drawing robust inferences regarding our findings, as it was not possible to conclusively discriminate between categorical and dimensional constructs (Ruscio et al., 2011).

Ambiguous results in taxometric analysis indicate that both models, categorical and dimensional, presented a comparable (good or bad) fit to the data, making conclusions about the latent structure of a construct hard to reach (Ruscio et al., 2011). Importantly, such ambiguity should not be interpreted as a lack of empirical information. Rather, when ambiguity is observed consistently across methods, samples, and analytic decisions, it constitutes meaningful evidence that the construct may not conform cleanly to either a categorical or a purely dimensional structure. In the present study, the ambiguous pattern of suicide ideation was consistently observed across different measurement instruments, samples, and sensitivity analyses. Importantly, these findings are unlikely to reflect methodological artifacts, as the analyses followed best-practice recommendations in the taxometric literature (e.g., consistency testing, simulation techniques, and the use of averaged CCFI values; Ruscio et al., 2007, 2010, 2018). This convergence across samples and analytic approaches suggests that the observed ambiguity is unlikely to be attributable to sample-specific characteristics, measurement limitations, or methodological artifacts. Instead, our findings are consistent with prior taxometric studies reporting ambiguous or divergent results for suicidal ideation and related constructs (e.g., Baptista et al., 2019; Haslam et al., 2020; Siddaway et al., 2021), and may reflect the inherent complexity of suicide ideation as a psychological phenomenon.

## Conclusion

If our assumption is correct, that is, the ambiguous findings are not due to measurements, samples, and statistical analyses, then it may be an inherent characteristic of the phenomenon, or at least of the way we are defining what suicidal ideation is. Suicidal ideation is a complex phenomenon encompassing multiple distinct characteristics (Reeves et al., 2022), such as passive thoughts of death, active desire for suicide, and preparatory or planning-related thoughts. The heterogeneity of these ideational components suggests that suicidal ideation may not be characterized by a single, uniform latent structure. When different ideational facets are examined jointly, they may reflect varying underlying structures, which could contribute to taxometric ambiguity. For example, some aspects of suicidal ideation, such as frequency or intensity of suicidal thoughts, may vary dimensionally, whereas other components, such as the emergence of active desire or preparatory ideation, may reflect more discontinuous shifts. Consequently, when suicidal ideation is treated as a unified phenomenon comprising multiple ideational components, taxometric analyses may fail to clearly favor either a categorical or dimensional model, resulting in ambiguous findings. Because suicidal ideation was treated as a unified phenomenon in our analyses, this may partly explain why our findings did not yield a clear latent structure.

Given the persistence of ambiguous and non-concordant findings regarding the latent structure of suicidal ideation, an alternative conceptual and analytic approach may be offered by the network perspective (Borsboom and Cramer, 2013; Borsboom et al., 2021). Network models allow discrete and continuous variables to be examined simultaneously and do not require assumptions about an underlying latent variable. From this perspective, suicidal ideation is conceptualized neither as a categorical nor as a dimensional phenomenon, but rather as a dynamic complex system in which symptoms and related characteristics mutually influence one another over time (Blanken et al., 2018). For a detailed discussion of the application of network analysis to suicidal ideation, see de Beurs et al. (2021).

Notwithstanding this study’s contributions, some methodological limitations should be considered when interpreting our findings. First, we performed this study with two convenient non-clinical samples, which can compromise the generalizability of our findings, as such samples may include relatively few individuals at elevated suicide risk. However, it is important to note that a substantial proportion of participants in both samples self-reported suicidal ideation and a history of suicidal behavior. Second, our data collection was conducted online, which can lead to a relatively high number of invalid and careless responses (Choi et al., 2017). Third, we used two specific measures of suicidal ideation (SIDAS and BSS) and a corresponding set of indicators, which were selected based on the researchers’ familiarity with these instruments. However, the choice of these measures was largely driven by convenience, and the use of alternative suicidal ideation instruments might have yielded different results. We suggest that future studies address these limitations and consider investigating individual components of suicidal ideation separately. In addition, researchers may benefit from applying alternative methodological approaches, such as network analysis, to better capture the complexity of this phenomenon.

Despite the highlighted limitations, we believe our study has two main strengths. First, although our findings do not provide conclusive evidence regarding the underlying nature of suicide ideation, they offer valuable insights for future investigations focused on its latent structure. Specifically, suicidal ideation may represent a complex phenomenon encompassing multiple components, each potentially characterized by different latent structures. Alternatively, conceptualizing suicidal ideation strictly as either categorical or dimensional may not be the most appropriate approach, and researchers may benefit from considering alternative frameworks for studying psychopathology. As such, the question of whether suicidal ideation is best understood as dimensional or categorical remains unresolved, underscoring the need for caution against premature conclusions favoring either a taxonic or dimensional interpretation. Second, our study appears to be the first, to our knowledge, to be conducted with adults in a Latin American sample, expanding results typically relying on White, educated, industrialized, rich, and democratic (WEIRD) samples. Most studies comprise samples from North America and Europe; these characteristics do not necessarily represent the world population (Azar, 2010). Therefore, our study also contributes to investigating the suicide ideation phenomenon in samples with different sociodemographic characteristics, demonstrating that the ambiguous results reported in the literature so far may not be related to these sample features.

## Data Availability

The raw data supporting the conclusions of this article will be made available by the authors, without undue reservation.
