# Supplementary material for: Persistently ambiguous: a taxometric investigation on two groups of suicidal ideation indicators
Source: Front Psychol. 2026 Feb 25;17:1729597. doi: 10.3389/fpsyg.2026.1729597 (PMC12975467; doi:10.3389/fpsyg.2026.1729597)
Supplement: Supplementary file 1 [file Table_1.docx]

**Supplementary Material**

Persistently ambiguous: a taxometric investigation on two groups of suicidal ideation indicators

Given that the decisions made in this study regarding the selection of valid indicators (SIDAS dataset) and the exclusion of participants based on screening items (BSS dataset) could potentially influence the results, we conducted sensitivity analyses to assess the robustness of our findings. Although these decisions were made to ensure data quality, their potential impact warranted additional examination.

For the BSS dataset, we first conducted a sensitivity analysis without excluding participants who scored 0 on both screening items. Based on the results of this analysis, we conducted an additional sensitivity analysis because the BSS indicators showed substantial deviations from normality, with both skewness and kurtosis exceeding 2. To address these deviations, we applied a base-10 logarithmic transformation using the log10() function in R. Because this transformation is undefined for zero values, we added one point to all BSS scores prior to transformation. For the SIDAS dataset, we conducted a sensitivity analysis without excluding the indicators that did not meet the effect-size criterion (d < 1.25; Items 1 and 2).

Descriptive statistics for all datasets are presented in Table 1. Table 2 reports the CCFI values for each taxometric method and dataset, as well as the corresponding mean CCFI values across methods. Figures 1, 2, and 3 illustrate the taxometric results for the MAMBAC, MAXEIG, and L-Mode methods for the three sensitivity analyses, respectively: (a) the full BSS dataset, (b) the BSS dataset following logarithmic transformation, and (c) the SIDAS dataset without excluding Items 1 and 2. Figures 4, 5, and 6 present the corresponding CCFI profiles for each dataset in the same order.

Overall, the results of the sensitivity analyses were consistent with the main findings and likewise supported an ambiguous latent structure. Although CCFI values derived from specific methods occasionally fell outside the ambiguous range, all mean CCFI values and aggregated CCFI values generated by the CCFI profiles remained within the 0.40–0.60 ambiguous interval. The sole exception was the BSS dataset following logarithmic transformation, for which the aggregated CCFI value from the CCFI profiles was 0.39, suggesting a dimensional structure. However, given that this result emerged only after data transformation, we consider it unlikely to reflect the true latent structure of the data.

**Supplementary Table 1.**

*Descriptive and quality of the indicators*

| BSS dataset (N = 2014) - Raw | | | | |
| --- | --- | --- | --- | --- |
|  | M (SD) | Skewness | Kurtosis | *d* |
| Wish for Death | 2.13 (3.51) | 1.61 | 1.53 | 4.90 |
| Preparation for Suicide | 0.73 (1.83) | 3.01 | 9.51 | 2.25 |
| Active Suicide Desire | 0.54 (1.29) | 2.96 | 9.15 | 2.33 |
| BSS dataset (N = 2014) - After log transformation | | | | |
|  | M (SD) | Skewness | Kurtosis | *d* |
| Wish for Death | 0.29 (0.40) | 0.94 | -0.75 | 4.86 |
| Preparation for Suicide | 0.12 (0.26) | 2.01 | 2.71 | 3.05 |
| Active Suicide Desire | 0.11 (0.22) | 1.94 | 2.66 | 3.52 |
| SIDAS dataset (N = 989) | | | | |
|  | M (SD) | Skewness | Kurtosis | *d* |
| Frequency of thoughts about suicide (SIDAS 1) | 4.91 (3.68) | 0.42 | -1.33 | 0.31 |
| Control over suicidal thoughts (SIDAS 2) | 5.53 (3.76) | -0.17 | -1.51 | -0.01 |
| Closeness to attempt (SIDAS 3) | 2.15 (3.16) | 1.29 | 0.23 | 2.75 |
| Distress (SIDAS 4) | 4.01 (4.07) | 0.42 | -1.52 | 2.25 |
| Interference with daily activities (SIDAS 5) | 3.19 (3.77) | 0.77 | -1.02 | 3.67 |

Note. BSS = Beck Scale for Suicide Ideation; SIDAS = Suicidal Ideation Attributes Scale; M = Mean, SD = Standard Deviation, d = Cohen’s d effect size.

**Supplementary Table 2.**

*Results of taxometric analysis*

|  | CCFI by method | | | | |
| --- | --- | --- | --- | --- | --- |
|  | | MAMBAC | MAXEIG | L-Mode | Mean CCFI |
| BSS dataset - raw | | 0.65 | 0.60 | 0.44 | 0.57 |
| BSS dataset - after log transformation | | 0.58 | 0.50 | 0.50 | 0.53 |
| SIDAS dataset (without items exclusion) | | 0.63 | 0.38 | 0.37 | 0.45 |

Note. BSS = Beck Scale for Suicide Ideation; SIDAS = Suicidal Ideation Attributes Scale; CCFI = Comparison Curve Fix Index; MAMBAC = Mean Above Minus Below a Cut; MAXEIG = Maximum Covariance; L-Mode = Latent Mode Factor Analysis.

**Supplementary Figure 1.**

Taxometric results BSS dataset - raw

**
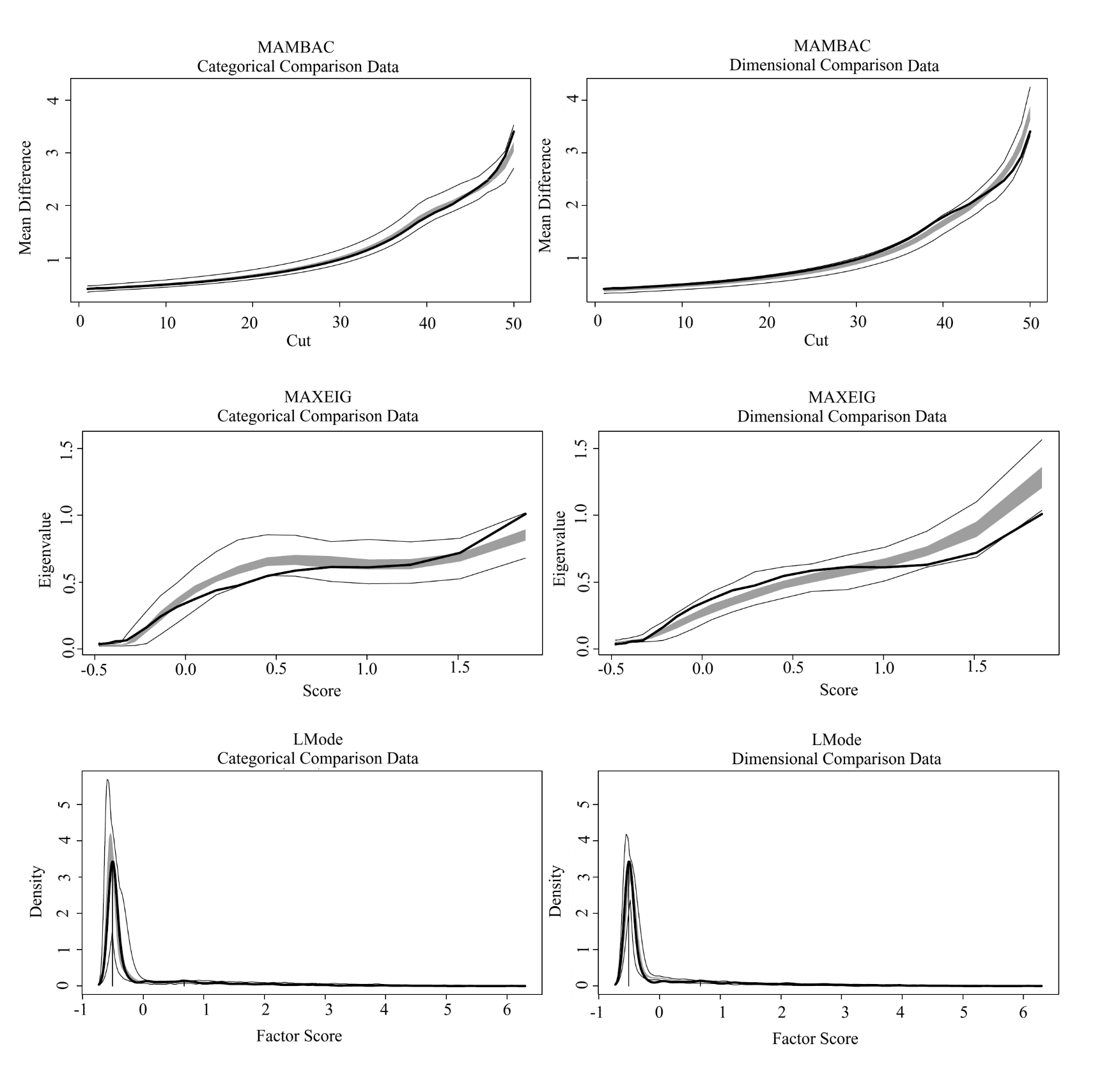
**

Note. BSS = Beck Scale for Suicide Ideation. The solid black line represents values computed from the observed data. The dark gray band represents values obtained from simulated taxonic (left panel) and dimensional (right panel) comparison data. Light gray lines represent variability across the simulated datasets. Base-rate estimates derived from each method were as follows: MAMBAC = 0.10, MAXEIG = 0.16, L-Mode = 0.44, and mean = 0.24. As expected under ambiguous solutions, estimates the derived base-rates estimates varied substantially across methods.

**Supplementary Figure 2.**

Taxometric results BSS dataset – after log transformation


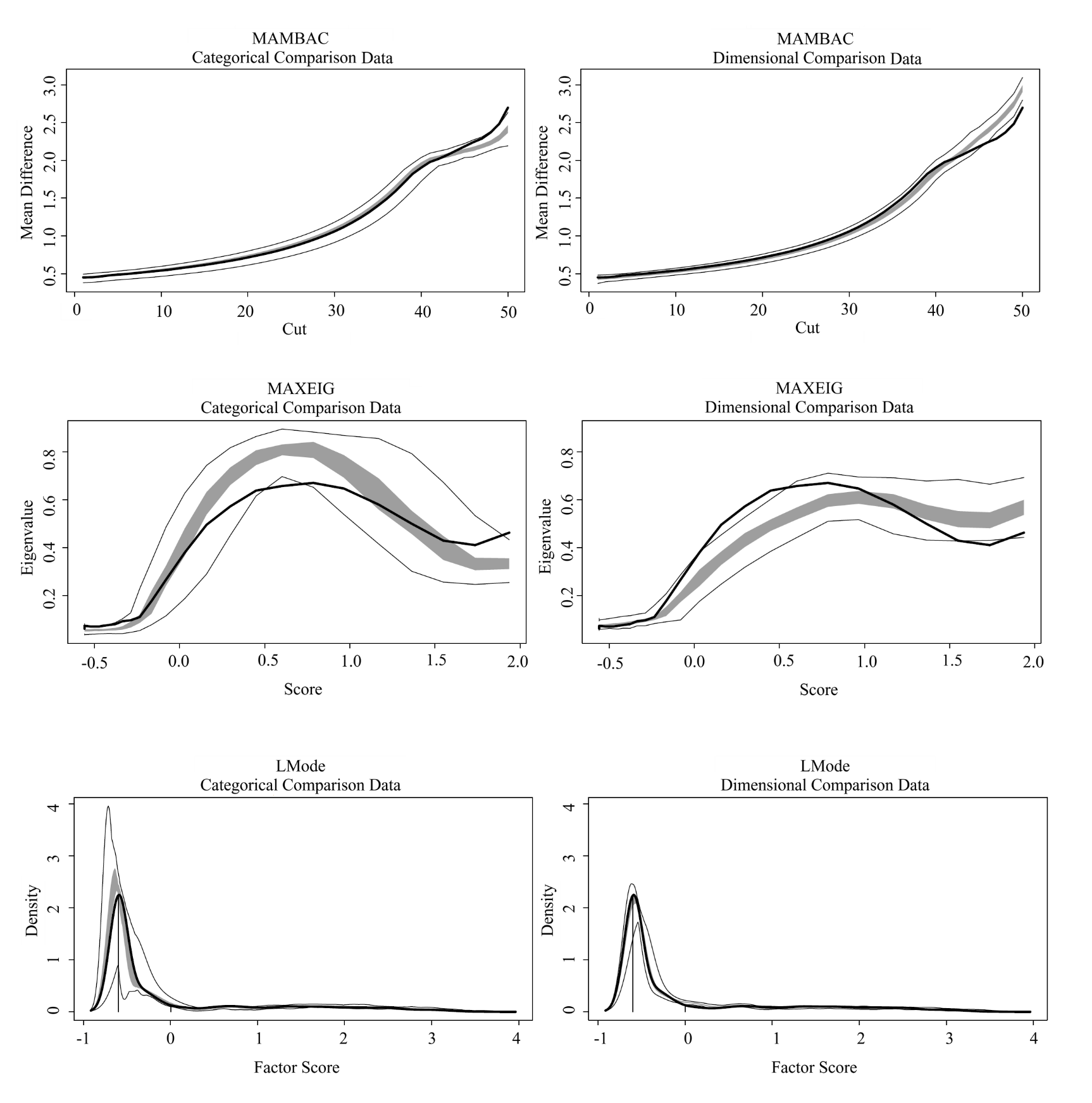


Note. BSS = Beck Scale for Suicide Ideation. The solid black line represents values computed from the observed data. The dark gray band represents values obtained from simulated taxonic (left panel) and dimensional (right panel) comparison data. Light gray lines represent variability across the simulated datasets. Base-rate estimates derived from each method were as follows: MAMBAC = 0.14, MAXEIG = 0.35, L-Mode = 0.63, and mean = 0.37. As expected under ambiguous solutions, estimates the derived base-rates estimates varied substantially across methods.

**Supplementary Figure 3.**

Taxometric results SIDAS dataset – without items exclusion


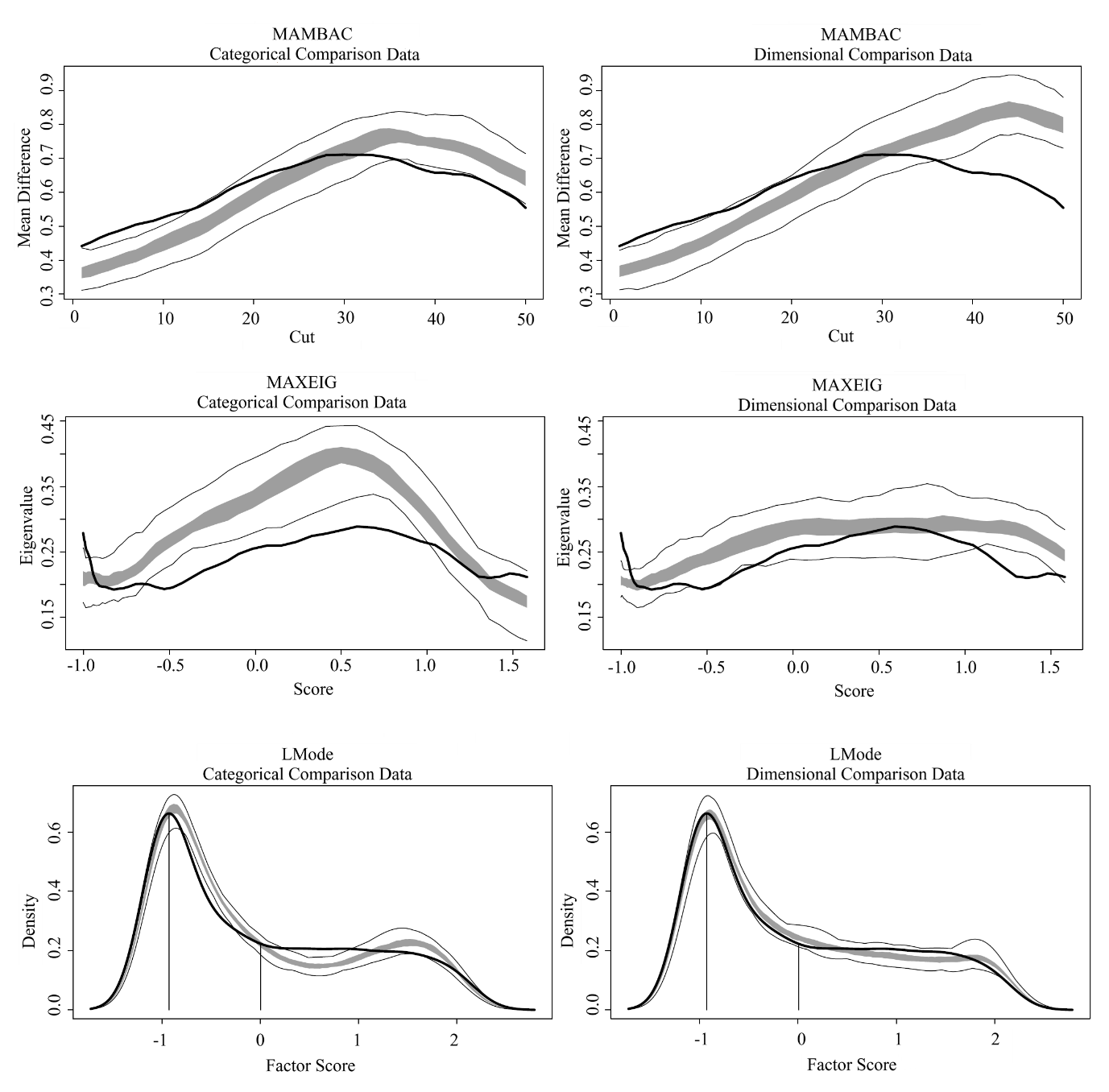


Note. SIDAS = Suicidal Ideation Attributes Scale. The solid black line represents values computed from the observed data. The dark gray band represents values obtained from simulated taxonic (left panel) and dimensional (right panel) comparison data. Light gray lines represent variability across the simulated datasets. Base-rate estimates derived from each method were as follows: MAMBAC = 0.01, MAXEIG = 0.87, L-Mode = 0.73, and mean = 0.54. As expected under ambiguous solutions, estimates the derived base-rates estimates varied substantially across methods.

**Supplementary Figure 4.**

CCFI profiles BSS dataset - raw


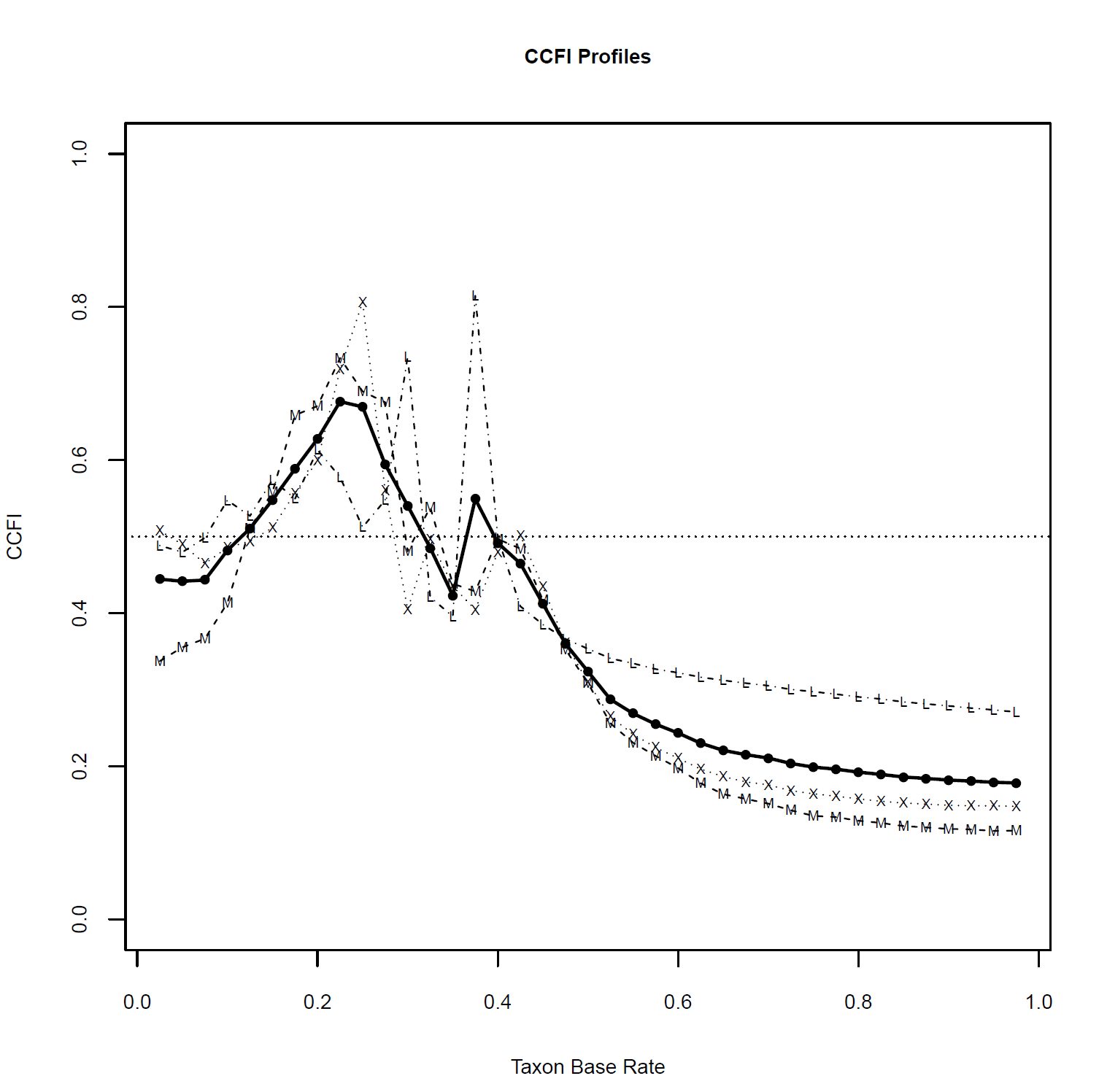


Note. BSS = Beck Scale for Suicide Ideation. CCFI = Comparison Curve Fix Index. CCFI profiles were generated across assumed taxon base rates ranging from 0.025 to 0.975 in increments of 0.025. Dotted lines with “M” represent CCFI values derived from MAMBAC, dotted lines with “X” represent CCFI values derived from MAXEIG, and dotted lines with “L” represent CCFI values derived from L-Mode. The solid black line represents the aggregated CCFI value across methods.

**Supplementary Figure 5.**

CCFI profiles BSS dataset - after log transformation


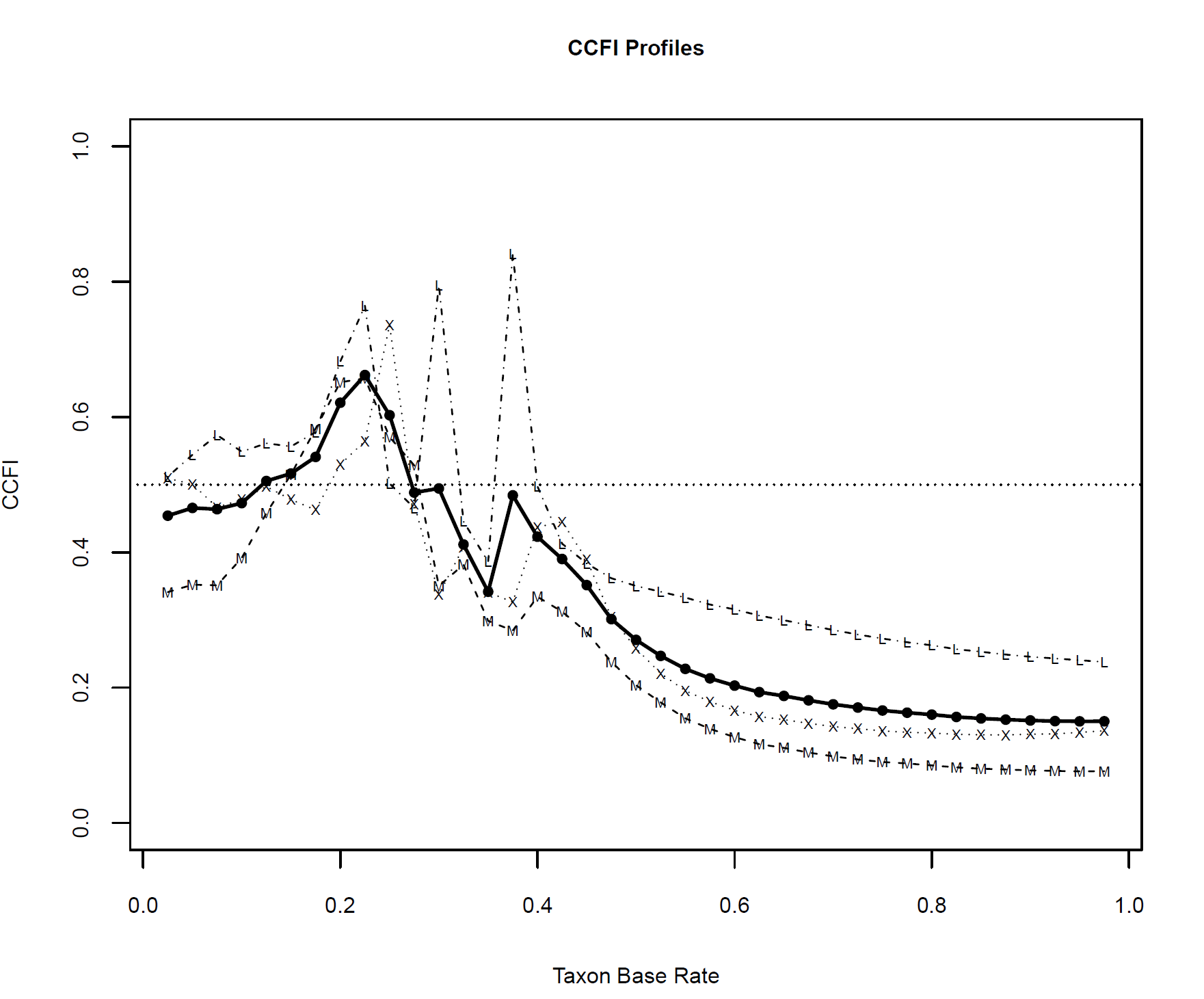


Note. BSS = Beck Scale for Suicide Ideation. CCFI = Comparison Curve Fix Index. CCFI profiles were generated across assumed taxon base rates ranging from 0.025 to 0.975 in increments of 0.025. Dotted lines with “M” represent CCFI values derived from MAMBAC, dotted lines with “X” represent CCFI values derived from MAXEIG, and dotted lines with “L” represent CCFI values derived from L-Mode. The solid black line represents the aggregated CCFI value across methods.

**Supplementary Figure 6.**

CCFI profiles SIDAS dataset – without items exclusion


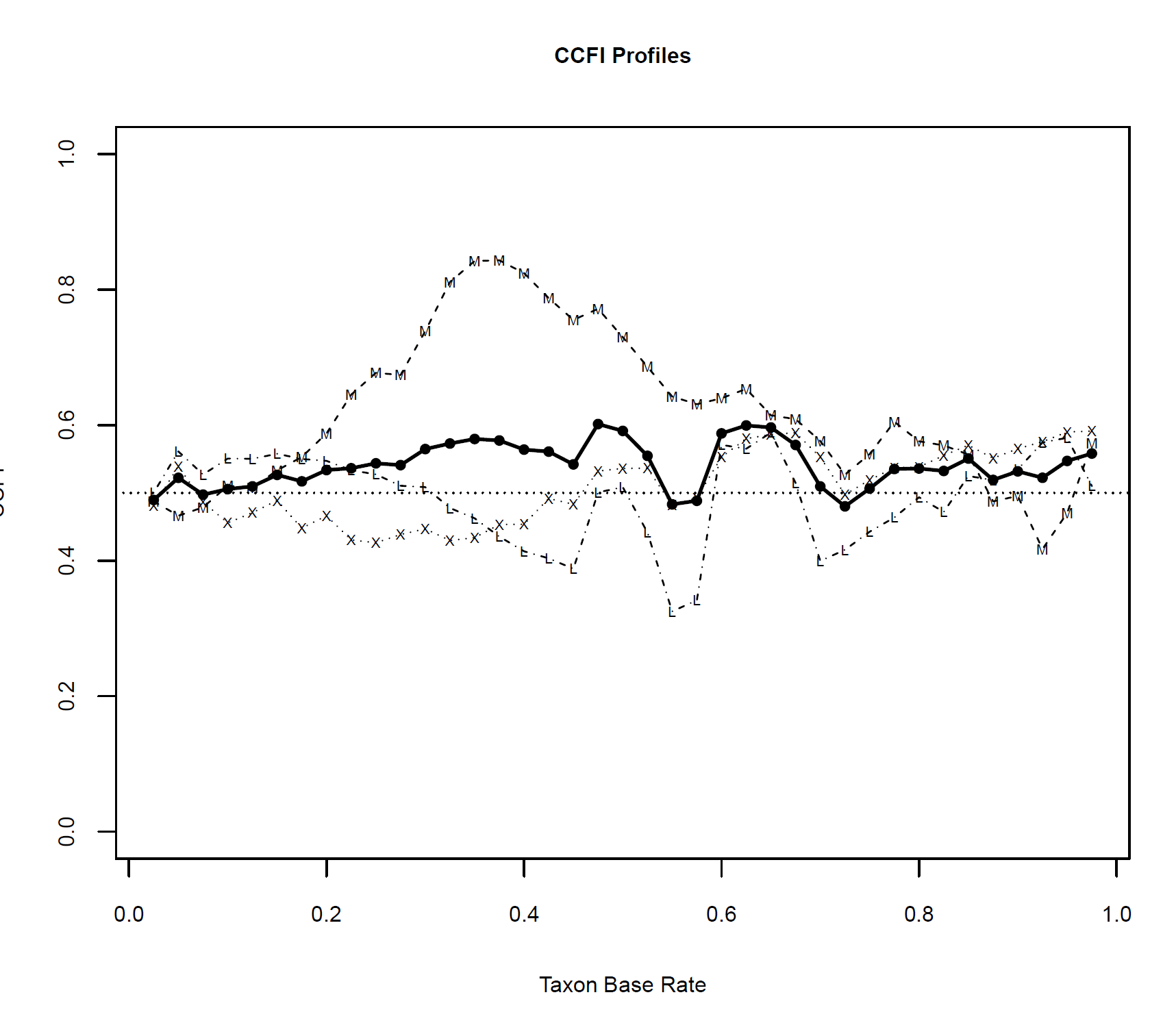


Note. SIDAS = Suicidal Ideation Attributes Scale. CCFI = Comparison Curve Fix Index. CCFI profiles were generated across assumed taxon base rates ranging from 0.025 to 0.975 in increments of 0.025. Dotted lines with “M” represent CCFI values derived from MAMBAC, dotted lines with “X” represent CCFI values derived from MAXEIG, and dotted lines with “L” represent CCFI values derived from L-Mode. The solid black line represents the aggregated CCFI value across methods.

**Supplementary Table 3.**

*Aggregated CCFI values generated by the CCFI Profiles*

|  | Aggregated CCFI by method | | | | |
| --- | --- | --- | --- | --- | --- |
|  | | MAMBAC | MAXEIG | L-Mode | Aggregated CCFI |
| BSS dataset - raw | | 0.42 | 0.41 | 0.46 | 0.43 |
| BSS dataset - after log transformation | | 0.33 | 0.36 | 0.47 | 0.39 |
| SIDAS dataset (without items exclusion) | | 0.67 | 0.53 | 0.49 | 0.55 |
